# A successful treatment of ganglionated plexi ablation for vagally mediated nocturnal atrioventricular block

**DOI:** 10.1016/j.jccase.2022.05.010

**Published:** 2022-06-10

**Authors:** Masanori Kobayashi, Tomohide Ichikawa, Yasushi Wakabayashi, Takashi Koyama, Hidetoshi Abe, Mitsuaki Itoh, Kohei Yamashiro

**Affiliations:** a Department of Cardiovascular Medicine, Matsumoto Kyoritsu Hospital, Matsumoto, Japan; b Department of Cardiovascular Medicine, Hyogo Brain and Heart Center, Himeji, Japan; c Department of Arrhythmia, Takatsuki General Hospital, Takatsuki, Japan

**Keywords:** Nocturnal bradyarrhythmia, Functional atrioventricular block, Catheter ablation, Ganglionated plexi

## Abstract

Patients suffering from sleep-related bradyarrhythmias are often underdiagnosed, due to the presence of asymptomatic cases. Although the consequence of increased nocturnal parasympathetic nerve activities and decreased sympathetic nerve activity during sleep are associated with nocturnal bradyarrhythmias, the detailed mechanisms are still unknown. It is well known that ganglionated plexi (GP) ablation is an effective therapeutic approach to modify autonomic nerve functions. Here, we report a case of successful treatment for the vagally mediated long ventricular pauses during sleep using autonomic modulation through GP ablation.

**Learning objective:**

Sleep-related bradyarrhythmias unrelated to sleep apnea or hypopnea are rare sleep disorders. Treatment of this disorder has not been established. High-frequency stimulation guided ganglionated plexi ablation could be an effective therapeutic approach to achieve long-term vagal attenuation to prevent vagally induced nocturnal bradyarrhythmias.

## Introduction

Sleep-related bradyarrhythmias unrelated to sleep-disordered breathing (SDB) were first described by Guilleminault et al. in 1984 [1] as sinus arrest observed during rapid eye movement sleep. Patients suffering from this disorder are often underdiagnosed, because nocturnal bradyarrhythmias in most cases are asymptomatic. Although the consequence of increased nocturnal parasympathetic nerve activities and decreased sympathetic nerve activity during sleep are associated with nocturnal bradyarrhythmias, the detailed mechanisms are still unknown. Effective therapeutic management has not been established, permanent pacemakers have been implanted in these patients, so far. It is well known that ganglionated plexi (GP) ablation is an effective therapeutic approach to modify autonomic nerve functions. However, it has not been fully investigated whether GP ablation could improve functional intermittent high-degree atrioventricular (AV) block and sinus node dysfunction to avoid unnecessary permanent pacemaker implantation. Here, we report an interesting case of successful treatment for the vagally mediated long ventricular poses during sleep using autonomic modulation through GP ablation guided by high-frequency stimulation (HFS).

## Case report

**Fig. 1:**
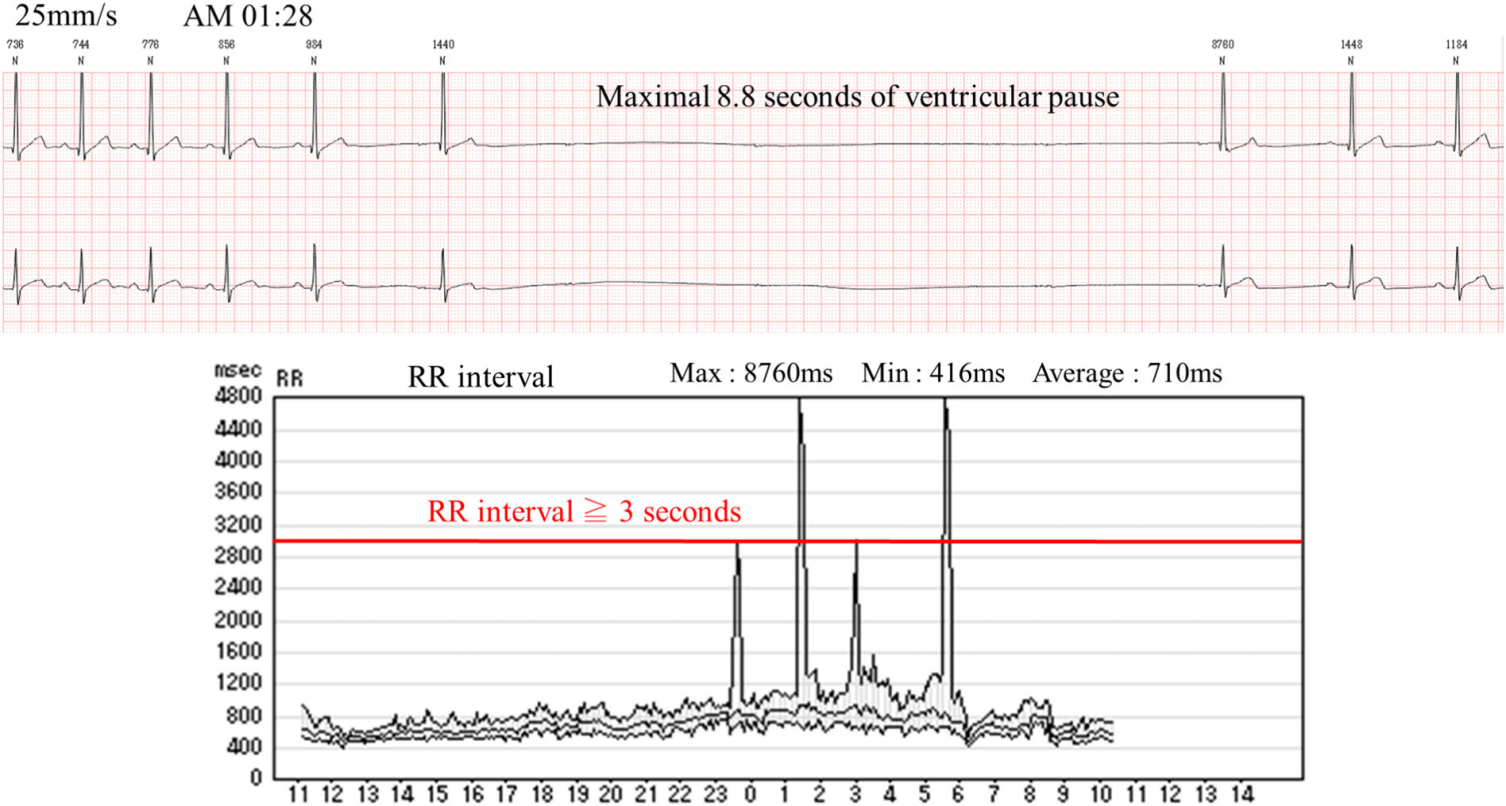
24-h Holter electrocardiogram shows an episode of vagally mediated paroxysmal atrioventricular (AV) block. A longest pause was characterized by a gradual slowing of the sinus rate (P—P interval) before and during the asystolic pause and a delay of AV conduction (prolonging PR) followed by complete AV block is typical of vagal origin.

A 33-year-old male with no significant past medical history was referred to our cardiovascular department due to palpitation. The patient did not experience daytime sleepiness, chest pain, presyncope, or syncope symptoms. He is a social drinker and smokes 20 cigarettes per day. He had no family history of sudden cardiac death (SCD). His body mass index was 23.8 kg/m^2^. Cardiac examination showed a regular heart rate of 64 beats per minute with normal heart sounds and no murmurs or rubs. The remainder of the physical examination was normal. The levels of serum electrolyte and thyroid function test were normal. Cardiac computed tomography was unremarkable. An electrocardiogram (ECG) showed normal sinus rhythm without any abnormalities in the QRS, ST segment, and QT interval. Atrial fibrillation (AF) was documented by previous ECG recording. A 24-h Holter ECG recording revealed intermittent sinus arrest and complete atrioventricular block during sleep, as well as 7 episodes of ventricular pause more than 3 s, with a maximal pause of 8.8 s at 1:28 AM. The longest pause occurred after the gradual decrease of the sinus rate (P—P interval) and the delay of AV conduction (prolonging PR) was shown before complete AV block occurred (Fig. 1). Polysomnography analysis, which was performed to evaluate SDB, showed no evidence of SDB with normal sleep structures and his apnea-hypopnea index was 3.0 events per an hour. Electrophysiological study showed that sick sinus syndrome and AV conduction disturbances were not observed. Neurally mediated syncope was excluded by the head up tilt table test. Transthoracic echocardiogram showed normal ejection fraction and no structural abnormalities.

According to these examination findings and the onset pattern of bradyarrhythmias, the possible mechanisms of bradyarrhythmic event in this case could be caused by vagal origin [2]. Because he did not experience any symptoms which were related to bradyarrhythmias or SDB, he was reluctant to accept pacemaker implantation. Considering his AF, we decided to undergo atrial GP ablation to reduce potential risks of abnormally prolonged ventricular pauses and modify the influential GP which may contribute to triggering and maintaining AF.

**Fig. 2:**
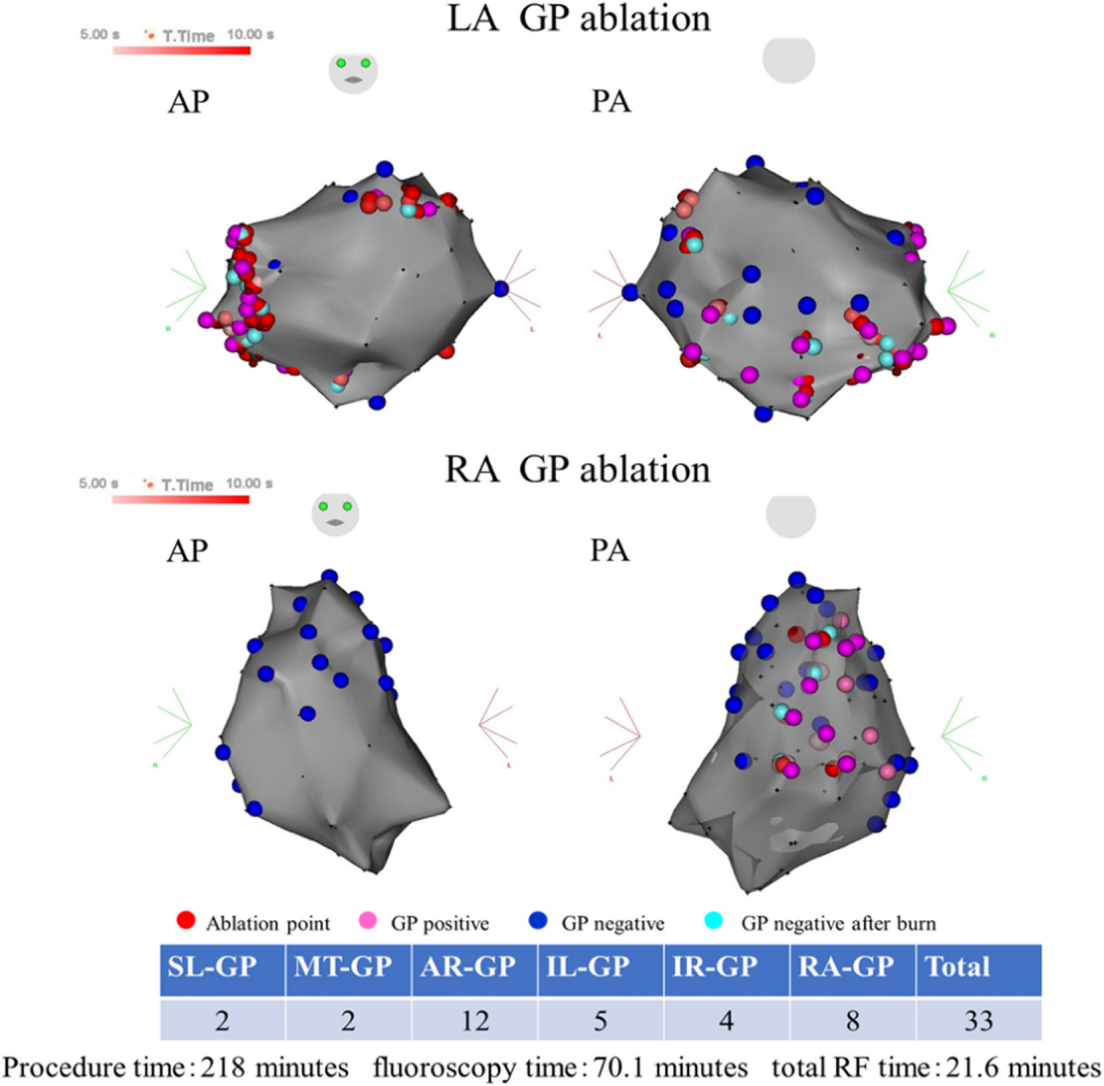
GP ablation site in both atria. The GP positive sites were tagged on the 3-dimensional map of both atria created by the CARTO system. GP ablation sites in the MTGP area was 2 points, SLGP area was 2 points, ILGP area was 5 points, ARGP area was 12 points, IRGP area was 4 points, right atrium was 8 points. AP, anteroposterior view; PA, posteroanterior view; GP, ganglionated plexi; LA GP, left atrial GP; MTGP, Marshall tract GP; SLGP, superior left GP; ILGP, inferior left GP; ARGP, anterior right GP; IRGP, inferior right GP; RAGP, right atrial GP.

After trans-septal puncture, the 3-dimensional (3D) both atria map was created using a 3.5-mm irrigated tip catheter (Navistar Thermo Cool; Biosense-Webster, Diamond Bar, CA, USA). The procedure was navigated by CARTO system (Biosense Webster, Diamond Bar, CA, USA). HFS was then delivered to the presumed parasympathetic ganglia sites with a frequency of 50 Hz, amplitude of 30 mA, and pulse width of 10 ms for 5 s per each site. A GP-positive site was defined as a site showing a vagal reflex defined as a prolongation of >50% of the R-R interval. The GP positive site was tagged on the 3D map of both atria created by the CARTO system. We identified the 5 major GP in the left atrium. The Marshall tract GP (MTGP) area is located within the fat pad anterior to the left superior pulmonary vein (PV) and left inferior PV (between the PVs and left atrium appendage). The superior left GP (SLGP) area is located on the roof of the left atrium, medial to the left superior PV. The inferior left GP (ILGP) area is located inferior to the left inferior PV. The anterior right GP (ARGP) area is located anterior to the right superior PV. The inferior right GP (IRGP) area is located inferior to the right inferior PV. After tagging the left atrium GP, we identified several GP in the right atrium. Multiple interconnections exist among the GP and there is a common final pathway to the AV node through the IRGP. Since the activated IRGP depress AV nodal conduction, ablation of the IRGP can lower the number and magnitude of GP activity on the left side. To minimize loss of vagal response, the GP sites were ablated in the sequence of MTGP, followed by SLGP, ARGP, ILGP, and IRGP, right atrial GP (Fig. 2). After each radio frequency (RF) application, HFS was repeated immediately at the ablation sites. If a positive parasympathetic response was still recorded, additional RF applications were delivered to the GP positive site. The end point of the procedure was inhibition of the parasympathetic response at each target after radiofrequency energy delivery. Sustained AF was induced by HFS but terminated to sinus rhythm during GP ablation. After the procedure, AF inducibility by HFS was diminished. It took a longer procedure time than expected. Therefore, we did not add PV isolation for AF. The procedure was finished without complications. The total RF application time was 21.6 min. The procedure time was 218 min and the radioscopy time was 70.1 min.

**Fig. 3:**
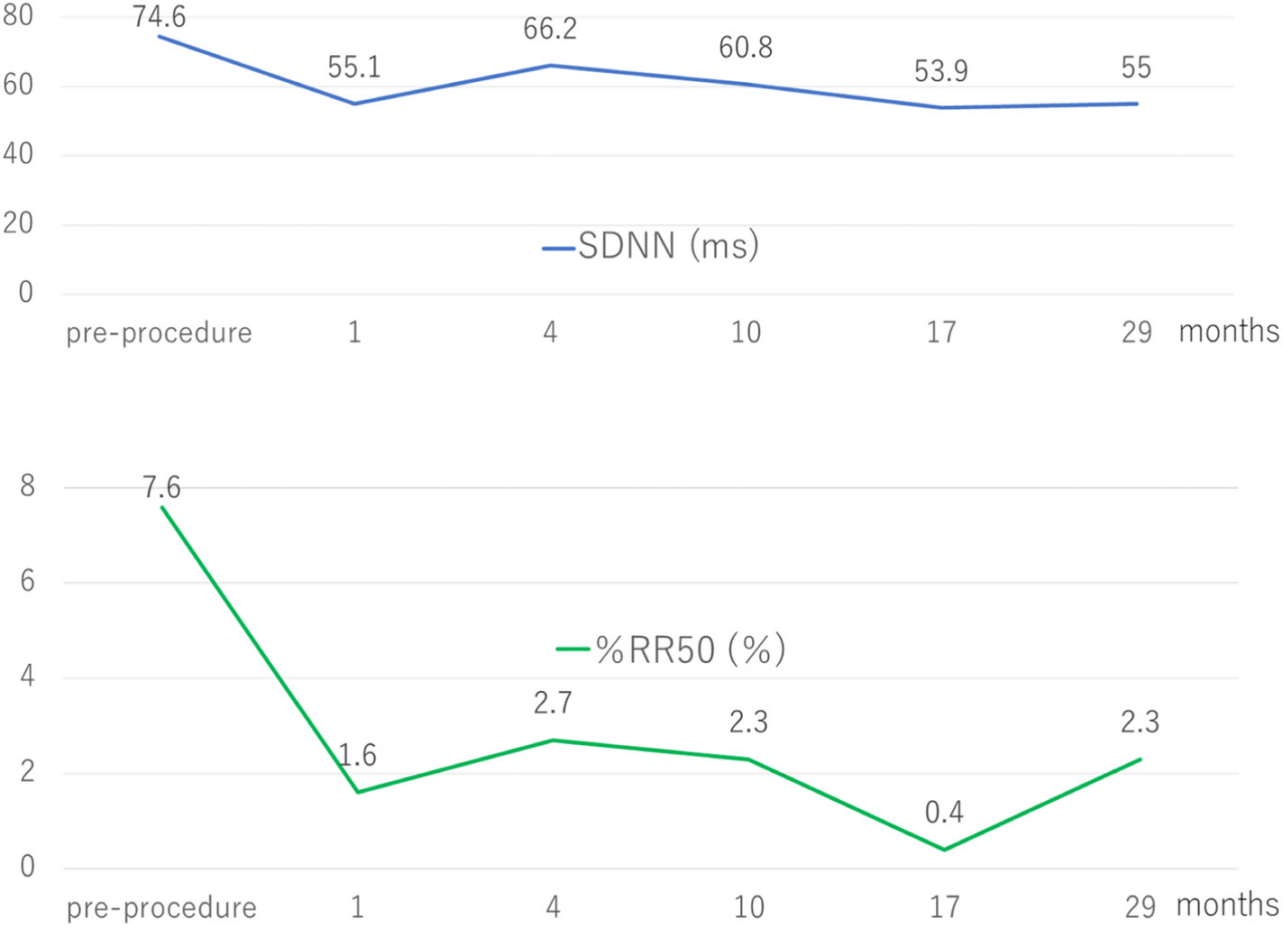
Heart rate variability. Compared Holter ECG recordings performed after the procedure with the pre-procedural measurement, SDNN and %RR50 were significantly decreased. SDNN, standard deviation normal to normal RR intervals; %RR50, percentage of successive R-R intervals that differ by more than 50 ms.

To evaluate the effect of GP ablation on bradyarrhythmias, implantable loop recorder (ILR; Reveal XT, Medtronic, Minneapolis, MN, USA) was implanted at the first postoperative day. Neither bradyarrhythmia (minimum heart rate < 30 beats per minute), nor long pause (RR interval > 3 s), nor AF was recorded by the ILR during 29 months follow-up period. 24-h Holter ECG recordings were performed before the procedure and at 1, 4, 6, 10, 17, and 29 months of follow-up, respectively. The remarkable changes in 24-h Holter ECG findings 1 month after GP ablation procedure were as follows: 1) Minimum heart rate was significantly elevated. 2) The episodes of ventricular pauses which last more than 3 s reduced from 7 to 0 times. 3) Standard deviation normal to normal RR intervals and percentage of successive R-R intervals that differ by more than 50 ms demonstrated significant decrease after the procedure (Fig. 3). These changes were preserved during the follow-up period. It was unclear whether his palpitation was derived from nocturnal bradyarrhythmias or not, but his symptoms disappeared after the procedure.

## Discussion

There currently is no standardized treatment for vagally mediated nocturnal bradyarrhythmias. Whether these bradyarrhymic events predispose to nocturnal SCD is still uncertain, but it is not possible to exclude the possibility that episodes of sinus arrests and AV blocks might become manifest for the first time with SCD. Although the benefit of pacemaker implantation for functional bradyarrhythmias is not yet established and there are published cases of those who have done well without the need for a pacemaker, cardiac pacing may decrease this potential risk [3], [4]. However, patients might experience disadvantages after pacemaker implantation. Considering his AF, GP ablation was recommended to modify the influential GP which may contribute to triggering and maintaining AF and nocturnal bradyarrhythmias.

It is well known that the intrinsic cardiac autonomic nervous system is composed of extensive epicardial neural networks of nerve axons, interconnecting neurons, and clusters of autonomic ganglia, which are known as GP. Almost all of GP are embedded within epicardial fat pads, the highest density of autonomic innervation is found at the posterior wall of the left atrium, particularly at the pulmonary vein-atrial junction [5]. These GP could contain several neuronal cell bodies that innervate the sinoatrial and atrioventricular nodes, thus controlling the parasympathetic innervation to the heart. Pachon et al. [6] reported cardiac autonomic modulation through catheter ablation guided by fast Fourier transformation analysis as an alternative treatment of functional bradycardias. Several other case reports and case series have also suggested that GP ablation is effective for patients suffering from neurocardiogenic syncope [7], [8], [9], [10], however, it is unclear whether GP ablation is effective for the patient with nocturnal bradyarrhythmias. In the present case, post-procedure 24-h Holter ECG and ILR recordings showed that GP ablation could be an effective therapeutic approach to achieve long-term vagal attenuation to prevent functional AV block and sinus bradycardia.

The potential complications associated with GP ablation are esophageal injury, phrenic nerve injury, cardiac perforation, thromboembolic event, and creating substrate for macro reentrant atrial tachycardia. Further studies are warranted to investigate the safety and utility of GP ablation in these conditions.

## Declaration of competing interest

The authors declare no conflict of interest.
